# ResSUMO: A Deep Learning Architecture Based on Residual Structure for Prediction of Lysine SUMOylation Sites

**DOI:** 10.3390/cells11172646

**Published:** 2022-08-25

**Authors:** Yafei Zhu, Yuhai Liu, Yu Chen, Lei Li

**Affiliations:** 1 College of Computer Science and Technology, Qingdao University, Qingdao 266071, China; 2 Dawning International Information Industry, Co., Ltd., Qingdao 266101, China; 3 Faculty of Biomedical and Rehabilitation Engineering, University of Health and Rehabilitation Sciences, Qingdao 266001, China

**Keywords:** SUMOylation, posttranslational modification, modification site prediction, deep learning, residual structure, machine learning

## Abstract

Lysine SUMOylation plays an essential role in various biological functions. Several approaches integrating various algorithms have been developed for predicting SUMOylation sites based on a limited dataset. Recently, the number of identified SUMOylation sites has significantly increased due to investigation at the proteomics scale. We collected modification data and found the reported approaches had poor performance using our collected data. Therefore, it is essential to explore the characteristics of this modification and construct prediction models with improved performance based on an enlarged dataset. In this study, we constructed and compared 16 classifiers by integrating four different algorithms and four encoding features selected from 11 sequence-based or physicochemical features. We found that the convolution neural network (CNN) model integrated with residue structure, dubbed ResSUMO, performed favorably when compared with the traditional machine learning and CNN models in both cross-validation and independent tests. The area under the receiver operating characteristic (ROC) curve for ResSUMO was around 0.80, superior to that of the reported predictors. We also found that increasing the depth of neural networks in the CNN models did not improve prediction performance due to the degradation problem, but the residual structure could be included to optimize the neural networks and improve performance. This indicates that residual neural networks have the potential to be broadly applied in the prediction of other types of modification sites with great effectiveness and robustness. Furthermore, the online ResSUMO service is freely accessible.

## 1. Introduction

The ubiquitin-like posttranslational protein modifiers are a family of proteins with structural similarity to ubiquitin that bind to the lysine side chains of target substrates through their C-terminal carboxyl groups. They include the small ubiquitin-like modifier (SUMO) proteins. Three SUMO proteins are predominant in mammalian cells: SUMO-1, SUMO-2, and SUMO-3, whereby the latter two are collectively termed SUMO-2/3 due to sharing 96% sequence identity and being indistinguishable by antibodies. SUMOylation of proteins is mediated through an enzymatic cascade [1]. SUMOylated proteins are predominantly localized in the nucleus, and SUMO regulates nuclear processes, including cell cycle control and DNA repair [2,3,4,5]. SUMOylation has been increasingly implicated in cancer, Alzheimer’s, and Parkinson’s diseases [6,7,8].

To understand the potential functions of SUMO on target substrates, it is essential to know which lysines are SUMOylated. Several purification strategies have been developed to study the global protein SUMOylome in a site-specific manner by employing epitope-tagged SUMO or SUMO antibodies [9,10,11,12]. Accordingly, thousands of exogenous or endogenous SUMOylated sites have been identified, and the vast majority correspond to SUMO-2/3. Based on these data, it is found that they reside within the consensus motif ΨKX[ED] or the inverted motif [ED]XKΨ, where Ψ is a large hydrophobic amino acid and X is any amino acid [12]. Nevertheless, many SUMOylation sites do not have consensus motifs [12].

Although purification strategies have greatly contributed to the study of SUMOylation, they have disadvantages such as high laboriousness and cost. By contrast, computational approaches for predicting SUMOylation sites have attracted considerable attention because of their convenience and efficiency. They include pSumo-CD [13], iAcet-Sumo [14], SUMOgo [15], SumSec [16], HseSUMO [17], SUMO-Forest [18], C-iSUMO [19], and iSUMOK-PseAAC [20], which are summarized in Table S1. They can be clustered into two groups: structure-based, which includes three classifiers, and sequence-based, which covers the remainder. In the structured-based group, HseSUMO [17] relies on four half-sphere exposure-based features [21], SumSec [16] combines bootstrap resampling, C4.5 decision tree, and majority voting with the two types of structural features predicted by SPIDER2 [22,23], and C-ISUMO [19] makes use of the AdaBoost algorithm combined with the sine and cosine of backbone torsion angles and accessible surface area predicted by SPIDER2 [22,23]. Because structural features of these classifiers are predicted based on protein sequences, the performance of the classifiers is influenced by feature prediction accuracy. Members of the sequence-based group can be further classified into two subgroups: machine-learning-based and deep-learning-based. The machine-learning-based subgroup includes four classifiers. pSumo-CD [13] incorporates the covariant discriminant algorithm with the feature of pseudo-amino acid composition (PseAAC). iAcet-Sumo combines the support vector machine (SVM) algorithm with One-Hot features [14]. SUMOgo integrates a random forest (RF) algorithm with consensus motif classification and features [15]. SUMO-Forest is a method based on cascade forest [24] with the features of bi-gram, k-skip-bigram, and statistics property [25]. The deep learning subgroup includes iSUMOK-PseAAC, an ANN (artificial neural network)-based classifier with the features of statistical moments and PseAAC [20]. The iSUMOK-PseAAC model is the latest in these reported classifiers and compares favorably to other classifiers [20]. The deep-learning algorithm, different from the traditional machine-learning algorithm that requires pre-defined informative features, has the strong capability to learn sparse representation in a self-taught manner and can auto-capture the most informative features. Therefore, deep-learning models perform better than traditional machine-learning models with big data [26].

These reported classifiers have made outstanding contributions to the study of SUMOylation. Nevertheless, the majority of them are inaccessible (Table S1). A second issue is that their dataset sizes are much smaller than the available data. For example, the largest dataset contains around 6000 SUMOylation sites, one-eighth of the available SUMOylation sites (~50,000). Data size is positively correlated with predictor performance [26,27]. Therefore, considering the state of big data, prediction models need to be re-examined for improvement.

In this study, we constructed a new SUMOylation dataset and performed sequence preference analysis. We further constructed and compared several in silico classifiers for predicting SUMOylation sites using this newly developed SUMOylation dataset. The classifiers combined various algorithms (e.g., RF, LightGBM, and convolution neural network (CNN)) and different encoding features (e.g., One-Hot and ZScale). We built a five-fold cross-validation dataset and independent dataset for model evaluation. We found that the classifier termed ResSUMO, which integrates a CNN based on the residual architecture with the ZScale encoding feature, performed better than other models based on cross-validation and independent tests. Furthermore, ResSUMO demonstrated better performance than the documented classifiers. Interestingly, ResSUMO, which contained five convolutional layers, compared favorably to CNN models with different convolutional layers, suggesting that the residual functions of the ResSUMO model could solve the potential degradation problem of multiple-layer neural networks and thus optimize them. Finally, we constructed the web server at http://bioinfogo.org/ResSUMO (accessed on 17 July 2022) to enable online prediction of human SUMOylation sites. We anticipate that accurate prediction by ResSUMO will facilitate the discovery of new SUMOylation sites and promote understanding of the pathogenesis and treatment of related diseases.

## 2. Materials and Methods

### 2.1. Data Construction

**Figure 1:**
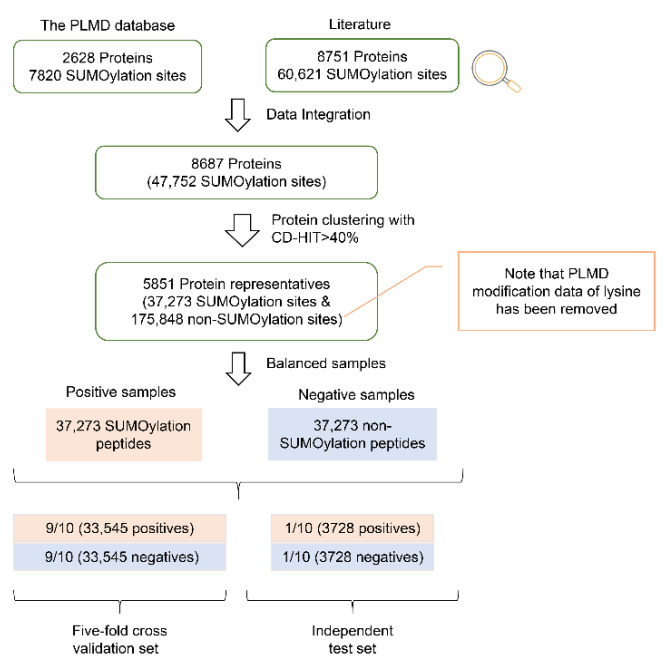
Schematic diagram of data collection and preprocessing for human SUMOylation datasets.

Figure 1 shows the process of dataset construction. Specifically, we collected 47,752 experimentally verified SUMOylation sites of 8687 proteins from the human proteome. These data are derived from either the literature or databases (Table S2) [11,20,28,29]. These proteins were grouped using the CD-HIT tool [28,29] with a sequence identity of 40%, and 5851 clusters were generated. The protein with the most significant number of SUMOylation sites in each cluster was considered representative. Toward this end, 5851 representatives were retained, corresponding to 37,273 SUMOylation sites. These SUMOylation sites were considered positive sites, and the remaining 217,568 lysine sites in these representatives were considered potential negative sites. We removed potential negative lysine sites that were annotated as having PTMs according to PLMD [30], leading to the retention of 175,848 lysine sites. Furthermore, we balanced positive and negative numbers by randomly selecting 37,273 lysine sites as the negative dataset, consistent with the approach of a previous study [31]. Additionally, we divided the data randomly into 10 groups: 9/10 (i.e., 33,545 positives and 33,545 negatives) as the cross-validation dataset and 1/10 (i.e., 3728 positives and 3728 negatives) as the independent test dataset. The dataframe.sample function in the pandas Python library was used for random number generation.

Because the performance of a prediction model is related to the length of input sequences, we evaluated different sequence lengths ranging from 15 to 41 with an increment of 2. The length of 39 was selected as it corresponded to the maximum area under the ROC curve (AUC) through five-fold cross-validation (Figure S1). Note that if the central lysine residue of the input sequence is located near the N-terminus or C-terminus of the protein sequence, “X” residues were added to the input sequences at the affected terminus to ensure the length was maintained.

### 2.2. Feature Encoding

Four different features were selected for model construction. They were Enhanced Amino Acid Composition (EAAC) [32], BLOcks SUbstitution Matrix 62 (BLOSUM62) [33], Amino Acid indices (AAindex) that include various physicochemical and biochemical properties of amino acids [34], and ZScale [32,35]. These encoding methods are described below.

#### 2.2.1. EAAC Encoding

EAAC encoding is based on amino acid composition (*ACC*) encoding, in which the frequencies of amino acid types in the peptide sequence are calculated. In EAAC encoding, the AAC value is calculated for a fixed-length sequence window (the default length being 5), continuously sliding from the N-terminus to the C-terminus of the peptide sequence (Figure S2) [32]. Therefore, each peptide sequence is encoded as a vector of 700 (=(39 − 5 + 1) × 20) items.

#### 2.2.2. BLOSUM62 Encoding

BLOSUM62 is the amino acid substitution matrix calculated from comparisons of sequences with a pairwise identity of no more than 62% (Figure S3). It has been widely used in constructing predictors of PTM sites, such as phosphorylation [36] and S-palmitoylation [37]. In this study, the peptide sequence with the length of 39 amino acids is encoded as a digital vector of 780 (=39 × 20) items.

#### 2.2.3. AAindex Encoding

AAindex is a public database of numerical indices representing various physicochemical and biochemical properties of amino acids and is widely used in bioinformatics research [34]. The AAindex database includes 544 amino acid properties. This study retained 531 properties after removing those with “NA”. As many of these properties are similar, we selected the nonredundant properties with the best prediction performance [38]. Accordingly, we calculated the AUC values of individual properties and ranked them in decreasing order. We then grouped the top-ranking properties ranging from 1 to 64 as a combined feature and calculated the AUC value. We found that the group of the first 14 properties had the largest AUC value (Table S3). As a result, the peptide sequence is represented as a 546 (=39 × 14) dimensional vector. Note that the AUC value was calculated using the RF algorithm for five-fold cross-validation. We encoded the peptide sequence filling character “X” as a zero vector of 14 dimensions.

#### 2.2.4. ZScale Encoding

In ZScale encoding, every amino acid type is characterized by five physicochemical descriptor variables (Table S4) [32,35]. Therefore, each peptide sequence is represented as a vector of 195 (=39 × 5) dimensions. The filling character “X” was encoded as a 5-dimensional zero vector.

## 3. Machine Learning Algorithms

### 3.1. Random Forest (RF) and Light Gradient Boosting Machine (LGBM)

The RF algorithm integrates multiple decision trees and chooses the classification with the most votes from the trees. There is no association between different decision trees in the forest; each tree depends on the values of a random vector sampled independently with the same distribution for all trees. To optimize the hyperparameters in the scikit-learn Python machine learning library, we used the “GridSearchCV” function, which could automatically adjust the number of decision trees, and finally determined the number of the trees as 140.

LGBM [39] is a robust gradient boosting framework that uses tree-based learning algorithms. This classifier was developed based on the Python module “sklearn”. Its hyperparameters were adjusted using the “GridSearchCV” function, similarly to RF.

### 3.2. The Architecture of the DL Models

#### 3.2.1. CNN-Based Classifiers

We constructed four one-dimensional CNN architectures with different encoding features (i.e., EAAC, BLOSUM62, AAindex, and ZScale), developed using Tensorflow. Each architecture contained four layers, described as follows (Figure S4).

1. Input layer. The peptide segment was converted into a two-dimensional matrix using each encoding feature listed above.
2. Convolutional layer. This layer includes two sequentially connected blocks. Each block includes a convolution sublayer, a batch normalization sublayer, a rectified linear unit (ReLU) [40] as the activation function, and a max-pooling sublayer. The number of convolution kernels was set to 128, and each convolution kernel size was set as 3. The max-pooling parameters pool_size, strides, and padding were set as 2, 1, and “same”, respectively.
3. Fully connected layer. The output from the above layer was flattened and received by the fully connected layer. This layer contained two fully connected sublayers, one with 128 neurons followed by another with 64 neurons.
4. Output layer. This layer contains a single neuron to output the probability score (within the range from 0 to 1), indicating the likelihood of the SUMOylation modification, which was calculated by the “Sigmoid” function as follows:
  (1) $$\mathrm{sigmoid}\left(x\right)=\frac{1}{1+e^{-x}}\;.\;$$

#### 3.2.2. The Residual Structure Layered CNN Architecture (RSCNN)

**Figure 2:**
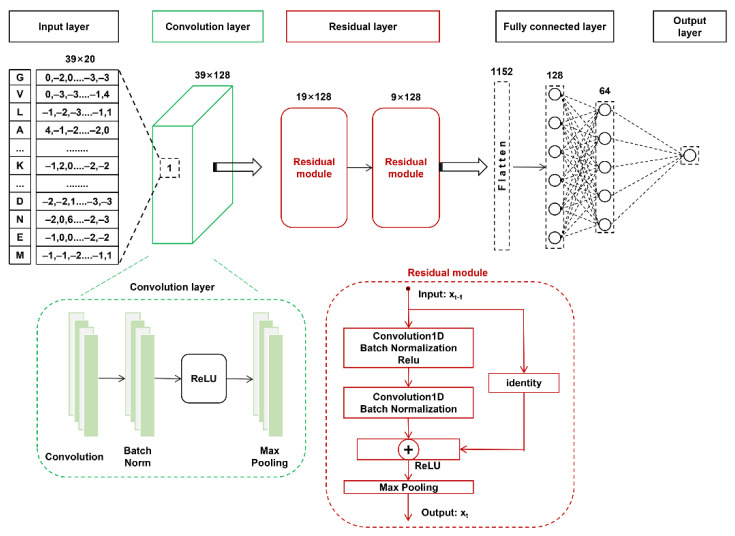
The architecture of the RSCNN model. The BLOSUM62 feature was used as the characteristic matrix of the input layer.

Based on the CNN model, RSCNN included an additional residual layer and made changes to the convolutional layer of CNN, described as follows (Figure 2).

1. Input layer. This layer is the same as the input layer of the above CNN architecture.
2. Convolution layer. This layer includes a single convolution sublayer, a batch normalization sublayer, a ReLU, and a max-pooling sublayer. The number of convolution kernels was 128, and the convolution kernel size was 3. The max-pooling parameters pool_size, strides, and padding were set as 2, 1, and “same”, respectively.
3. Residual layer. This layer contains two sequentially connected residual module blocks. Each block includes two convolution sublayers and two batch normalization sublayers. In each convolution sublayer, the number of convolution kernels, convolution kernel size, and the convolution parameter padding were 128, 3, and “same”, respectively. The output $x_{t}\;$ of the residual module block t can be calculated using the following formula:
  (2) $$x_{t}=P\left(R\left(x_{t-1}+\delta \left(x_{t-1},w_{t}\right)\right)\right)$$
  where $x_{t-1}$ is the input of the *t-*th residual module block; $w_{t}$ represents a set of weights of the *t-*th block. δ refers to the convolution and batch normalization operation in the *t-*th block, *R* is the ReLU activation function, and *P* is the max-pooling function.
4. Fully connected layer. This layer is the same as for the above CNN architecture.
5. Output layer. This layer is the same as for the above CNN architecture.

### 3.3. The Strategy to Avoiding Overfitting for the DL Approaches

In these DL architectures, the dropout rates of the neuron units in the convolution sublayer were 0.5; the L2 regularization of the neuron units in the full connection sublayer was 0.01; the hyperparameters were optimized using the Adam algorithm based on the binary cross-entropy function. The maximum training period was set as 500 epochs. In each epoch, the training dataset was separated and iterated in the batch size of 512, and the prediction accuracy was calculated using the validation dataset. The early stopping optimization technique was used to avoid overfitting, where the training process was stopped when the validation accuracy did not increase after 50 iteration cycles. Finally, the hyperparameters with the best accuracy were retained. Figure S5 shows the training and validation accuracy and loss curves of the ResSUMO model for five-fold cross-validation.

### 3.4. Performance Evaluation Strategies

We used four statistical measures (e.g., sensitivity (*Sn*), specificity (*Sp*), accuracy (*ACC*), and Matthew’s correlation coefficient (*MCC*)) to evaluate the performance of the predictive model, defined as follows:

(3) $$Sn=\frac{TP}{TP+FN}\;$$

(4) $$Sp=\frac{TN}{TN+FP}$$

(5) $$ACC=\frac{TP+TN}{TP+FP+TN+FN}$$

(6) $$MCC=\frac{TP\times TN-TN\times FP}{\sqrt{\left(TP+FN\right)\times \left(TN+FP\right)\times \left(TP+FP\right)\times \left(TN+FN\right)}}\;$$

where *TP*, *TN*, *FP*, and *FN* are the number of true positives, true negatives, false positives, and false negatives, respectively. In addition, the prediction performance was measured using the receiver operating characteristic (ROC) curve and the area under the ROC curve (AUC). Generally, the closer the AUC value to 1, the better the model’s prediction performance.

### 3.5. Statistical Analysis

A paired Student *t*-test was used to evaluate the statistical difference in the means between the two populations.

## 4. Results

### 4.1. Construction of the SUMOylation Dataset and Sequence Preference Analysis

We collected 47,752 experimentally verified SUMOylation sites on 8687 human proteins from the literature and the PLMD database [11,20,28,29] (see Table S2 for details). After CD-HIT clustering, 37,273 SUMOylation sites of the 5851 protein representatives as positive samples (See Methods for details).

A lysine residue may undergo different types of PTM, e.g., ubiquitylation and SUMOylation [41]. We reason that the lysine sites experimentally verified for any type of PTM are more likely to be SUMOylated than those without any PTM annotation. In other words, the lysine sites annotated with PTMs may be unsuitable for use as negative samples in our study. Therefore, we removed such lysine sites annotated with PTMs in the PLMD database from the representatives and kept the remaining 175,848 lysine sites as negative samples. As the number of the negative sites was significantly larger than that (37,273) of the positive sites, we balanced the data by randomly selecting the same number (37,273) of negative sites, consistent with previous studies [31].

Both positive and negative samples were randomly separated into two groups: 9/10 as the cross-validation dataset and the rest 1/10 as the independent test dataset (Figure 1). To the best of our knowledge, our dataset covers many more SUMOylation sites than the reported SUMOylation datasets used for prediction modeling, the largest of which contains 5963 SUMOylation sites (see Table S1 for details). Because a predictor’s performance is related to the input peptide length, we evaluated different peptide lengths within the range from 15 to 41 with an increment of 2. We selected the length of 39, which had the largest corresponding AUC value following five-fold cross-validation (Figure S1).

**Figure 3:**
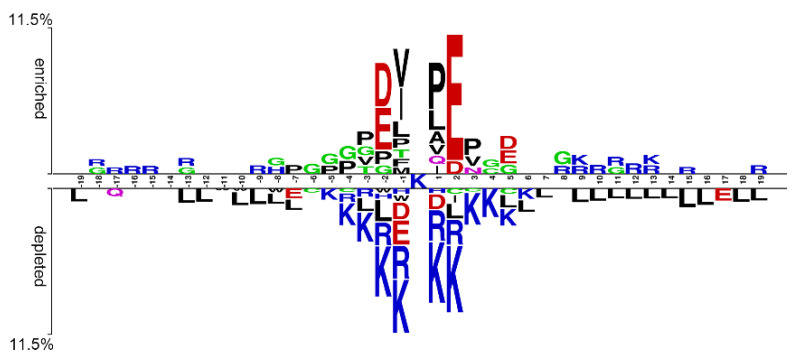
Sequence pattern surrounding the SUMOylation sites, including the significantly enriched and depleted residues based on SUMOylation-containing peptides and nonmodified peptides in our dataset (*p* < 0.05, *t*-test with Bonferroni correction). The pattern was generated using the two-sample logo method (Vacic et al. 2006).

We examined the patterns and conserved motifs of SUMOylation-containing peptides by comparing positive and negative samples in our dataset using the two-sample logo method [42]. Figure 3 shows that the residues located at the −2, −1, +1, and +2 positions (i.e., P − 2 to P2) have noticeable features compared with those at other positions. Specifically, the positively charged amino acids (e.g., K and R) are depleted at P − 2 to P2. By contrast, the negatively charged amino acids (e.g., D and E) are enriched at the P − 2 and P2 but depleted at P − 1 and P1. Hydrophobic amino acids (e.g., V, A, L, P) are enriched at P − 1 and P1. These observations are in agreement with previous results [12,20].

### 4.2. Performance Evaluation of Various Classifiers Combined with Distinct Features

Machine learning algorithms are commonly used in the field of PTM site prediction and demonstrate good performance, including random forest (RF) [43,44], support vector machine (SVM) [45], and naive Bayes (NB) [46,47]. Additionally, a light gradient boosting machine (LGBM) is also widely used with outstanding performance [48,49] due to the advantages of fast training speed and high efficiency, accuracy, and capability to handle large-scale data [49,50]. Recently, deep learning (DL) algorithms (e.g., convolutional neural network (CNN)) have demonstrated superior prediction performance in the field of bioinformatics, such as in the prediction of modification sites on DNA, RNA, and proteins [26,27,31,51,52,53] and many domains of social relevance [54,55,56,57,58,59,60,61,62].

Additionally, the deep residual learning algorithm, which was reported in 2015 [46,63] and has been used in computer vision problems (e.g., image classification [64] and target detection [65]), has shown great success in the prediction of protein–protein interactions [66,67] and DNA–protein binding [68]. Therefore, we attempted to examine the CNN algorithm integrated with residual learning (RSCNN). In summary, we chose four different algorithms (i.e., RF, LGBM, CNN, and RSCNN) to construct models for predicting SUMOylation sites.

Besides algorithms, prediction models are based on encoding features, and their prediction performance is affected by these features. We examined 11 common sequence-based or physicochemical features using the iLearnPlus tool [69] (Table S5). The four features showing the best performance for SUMOylation site prediction (i.e., EAAC, BLOSUM62, AAindex, and ZScale) were selected for subsequent study (Table S5). Accordingly, we constructed sixteen models covering four algorithms (i.e., RF, LGBM, CNN, and RSCNN) combined with four distinct encoding schemes (i.e., EAAC, BLOSUM62, AAindex, and ZScale).

**Figure 4:**
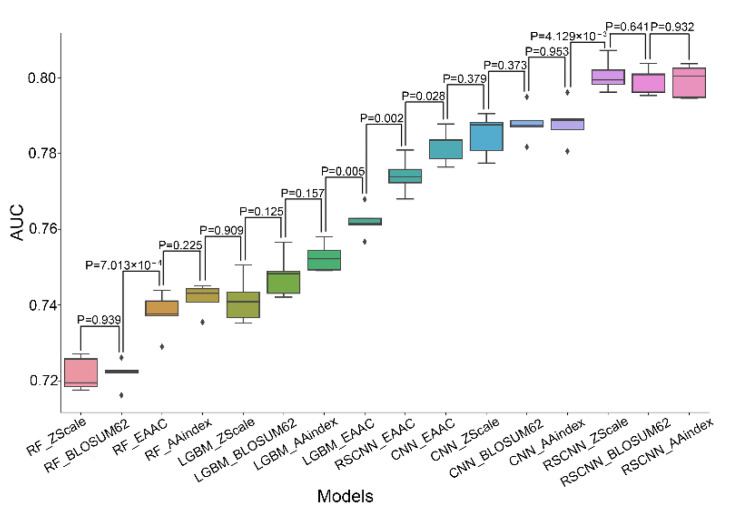
AUC values of different classifiers in terms of five-fold cross-validation. The classifiers are displayed in ascending order of AUC values. The statistical differences between the neighboring classifiers were calculated using paired Student *t*-test. The name for each model is a combination of the names of the involved algorithm and the encoding feature.

The performance of the sixteen models was evaluated based on five-fold cross-validation (Figure 4 and Figure S6). Interestingly, the performances were mainly determined by the algorithms rather than features. The RF-based models had the lowest performance (average AUC: 0.731 ± 0.009), followed by the LGBM-based models (average AUC: 0.751 ± 0.008) and the CNN-based models (average AUC: 0.786 ± 0.002), and the RSCNN-based models had the most outstanding performance (average AUC: 0.793 ± 0.011). By contrast, the models with different features had similar performance, e.g., the average AUC value of EAAC-based models was 0.764, and that of the ZScale-based models was 0.762. These observations indicate that algorithms have a greater impact on model performance than features in terms of predicting SUMOylation sites. Despite this, features have effects on prediction performance to some extent. For example, the model constructed using the EAAC feature had the lowest AUC value in the RSCNN-based models, whereas it had the highest AUC value in the four LGBM-based models (Figure 4 and Figure S6, and Table 1).

### 4.3. Most RSCNN Models Compare Favorably to Other Models

In the sixteen models, the RSCNN models with either of the three features (i.e., AAindex, BLOSUM62, and ZScale) had similar mean AUC values (~0.8; *p*-value > 0.641, Student’s *t*-test), and their AUC values were significantly larger than those of the remaining classifiers (*p* < 4.129×10^-3^, Figure 4). They also showed the best performance for SN, *ACC*, and *MCC* when the SP value was fixed as 0.650 (Table 1). For example, their average *MCC* values were around 0.457, whereas those of the remaining classifiers were below 0.438, and their *ACC* values (around 0.726) were larger than those (<0.717) of the other models (Table 1). Therefore, the RSCNN models with any of the three features had the best performance in terms of five-fold cross-validation.

We evaluated the performance of the sixteen models using the independent test dataset (Figure S7 and Table S6). The three best RSCNN models identified in cross-validation still showed similar performance and were superior to the remaining models (Figure S7). Therefore, these three models performed best in cross-validation and independent tests. Of the three features, ZScale encoding corresponds to a vector of 195 dimensions, smaller than BLOSUM62 (represented as a 780-dimensional vector) and the AAindex (represented as a 546-dimensional vector). Since ZScale requires the least memory of the three features, we chose an RSCNN architecture with the ZScale scheme for the final model for predicting SUMOylation sites, dubbed ResSUMO. It should be noted that the performance of the sixteen models in cross-validation was similar to those for the independent test, suggesting that these models were not overfitting (Figure S8).

### 4.4. Our Model ResSUMO Shows Superior Performance to the Reported Classifiers

Eight SUMOylation predictors have been documented so far, summarized in Table S1. Among them, the most recent two predictors were reported to have the best performance, i.e., SUMO-Forest [18] and iSUMOK-PseAAC [20]. Therefore, we compared them with ResSUMO to evaluate the performance of ResSUMO in prediction. SUMO-Forest [18] is a cascade forest-based classifier constructed using 755 positive and 9944 negative samples; iSUMOK-PseAAC was developed based on 4987 positives and 5000 negatives [20]. Both models are currently inaccessible, so we reproduced them by referring to the literature. Table S7 shows that the reproduced models had similar performance to the documented models, indicating that the former could recapitulate the latter. Therefore, the reproduced SUMO-Forest and iSUMOK-PseAAC models were used for subsequent study.

We estimated the performance of the reproduced SUMO-Forest and iSUMOK-PseAAC in our independent test data (i.e., 3728 positive and 3728 negative sites). Surprisingly, their AUC values are around 0.55, significantly lower than that (0.801) of ResSUMO (Table S8). We generated the two sample logo plots using their original datasets to determine why the AUC values were so low. Figure S9 shows that E@P2 is significantly enriched in the SUMOylated peptides, and its enriched value is 61.3% for the SUMO-Forest dataset or 23.8% for the iSUMOK-PseAAC dataset. By contrast, the enriched value of our dataset is only 10.0% (Figure 3). Compared with the two-sample logo of our dataset, their datasets are biased to the consensus motif ΨKX[ED] but have few peptides with the inverted motif [ED]XKΨ, which may be responsible for the models’ poor performance in our independent test dataset. These observations highlight the necessity of using a large-scale dataset for model development, as such a dataset will cover the majority of modification characteristics.

**Figure 5:**
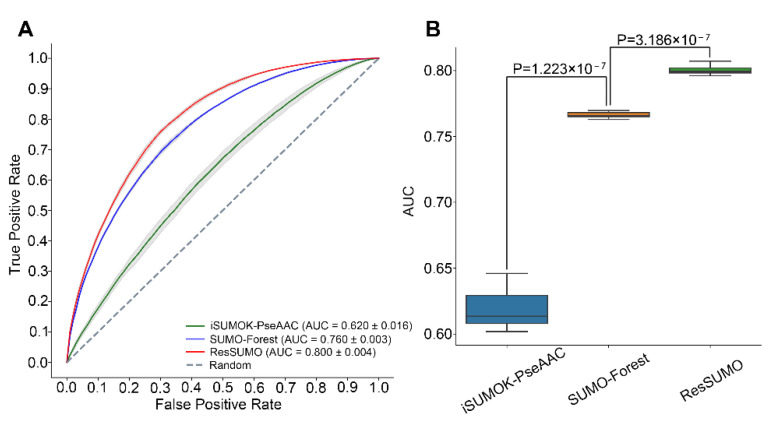
The AUC values of ResSUMO, the reproduced SUMO-Forest, and iSUMOK-PseAAC in five-fold cross-validation (**A**) and performance comparison (**B**).

To fairly compare the three predictors, we re-trained and evaluated SUMO-Forest and iSUMOK-PseAAC using our dataset in five-fold cross-validation. Figure 5 shows that the mean AUC values of ResSUMO, SUMO-Forest, and iSUMOK-PseAAC are 0.800, 0.760, and 0.620, respectively, and the value for ResSUMO is significantly larger than for these two models (*p* < 3.186×10^−^^7^, paired *t*-test). Similarly, ResSUMO demonstrated better performance in the independent test (Table 2). Therefore, ResSUMO compares favorably to the reported classifiers. Its superior performance can be explained according to the following two primary reasons: the deep learning model of ResSUMO improves the effectiveness and generalization because a large-scale training dataset is used; the idea of residual structure effectively captures the characteristics of the SUMOylation sites, which proved to be an effective strategy.

### 4.5. Construction of the Online SUMOylation Predictor

**Figure 6:**
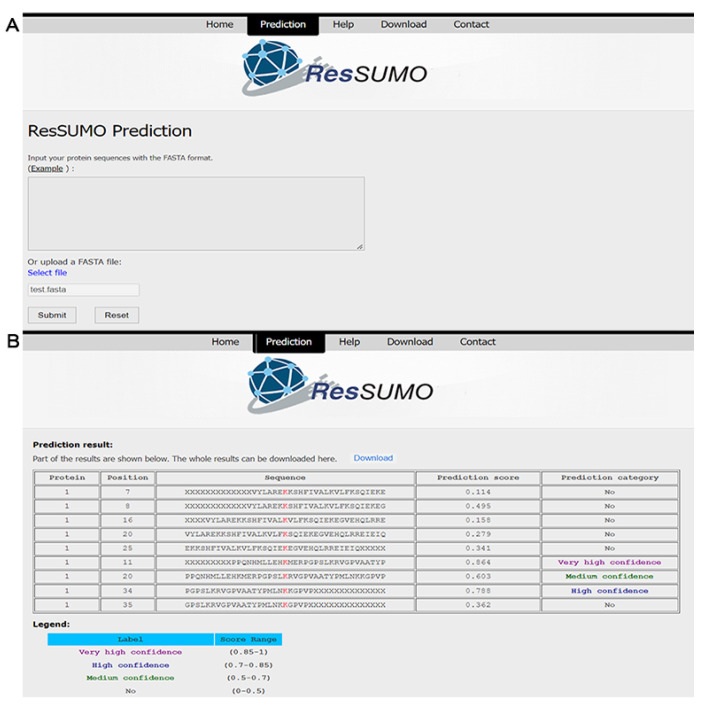
The online ResSUMO interface for the prediction of lysine SUMOylation sites (**A**) and its application in prediction (**B**).

We developed an easy-to-use online tool for predicting human SUMOylation sites via http://bioinfogo.org/ResSUMO (accessed on 17 July 2022). The users could directly input the query protein sequences in FASTA format or upload the sequence file (Figure 6A). After job submission, the prediction will start, and the process may take a few minutes. Finally, the prediction results are output in tabular form with five columns: protein, position, sequence, prediction score, and prediction category (Figure 6B). The predicted results can also be downloaded as a data file.

## 5. Discussions

Various predictors have been developed for predicting SUMOylation sites. The early predictors (e.g., pSumo-CD [13], HseSUMO [17], and SUMO-Forest [18]) were built using small datasets, including only 755 human SUMOylation sites, due to the limited availability. Later, iSUMOK-PseAAC [20] was constructed by expanding the dataset, covering 4987 positive samples. Recently, 40,765 and 14,869 SUMOylation sites on human proteins were found in different studies [11,70]. We used some newly identified sites as an independent test dataset to examine the documented predictors, which had poor performance (AUC: around 0.55; Table S8). This suggests that the limited dataset cannot cover all features of SUMOylated peptides. Indeed, we reconstructed them using our large dataset, and their performance increased significantly (Table 2). Therefore, it is essential to use a large dataset to build a reliable predictor.

In this study, we constructed 16 models based on four different algorithms and four distinct encoding features. We compared these models and found that feature selection affected model performance, and algorithm selection also significantly impacted the performance. Therefore, exploring various algorithms is necessary for modeling. We also found that the model ResSUMO, based on an improved CNN method with residual structure, achieved the best performance in the 16 models and was demonstrated to be superior to the documented classifiers in both cross-validation and independent tests. Specifically, the ResSUMO model had better performance than the CNN models.

Convolutional neural networks have led to breakthroughs in image classification and PTM site prediction [71,72,73]. The networks naturally integrate features and classifiers in a multilayer fashion, and the features can be enriched by the number of stacked layers (depth). The network depth is crucial, and the prediction performance greatly benefits from using relatively deep models. However, with the increase in network depth, the accuracy is usually saturated and degrades rapidly, indicating that deeper neural networks are more challenging to optimize [63]. Indeed, we constructed CNN models with different numbers of convolution layers ranging from 1 to 8 and compared their performance in five-fold cross-validation. Table 3 shows that the AUC values of the CNN models increased from a single convolution layer to two convolution layers but decreased with additional layers. To solve the potential degradation problem, a residual learning framework has been developed to jump over some convolution layers and ease the training of deeper networks [63]. In this study, we constructed a residual learning-based CNN model ResSUMO that contains five convolution layers (Figure 2). ResSUMO compared favorably to the CNN models with different convolution layers (Table 3). This indicates that the residual structure in the deep CNN model could allow optimizing the neural networks for SUMOylation site prediction.

## 6. Conclusions

In this work, we constructed a new SUMOylation dataset and numerous models integrating different algorithms and encoding features. Of these, the deep residual learning-based model ResSUMO demonstrated the best performance. Although ResSUMO improved performance in the prediction of protein SUMOylation sites, there are several proposals for further improvement. Firstly, in this study, we preliminarily explored the application of a deep residual learning algorithm, but further investigation is necessary for prediction studies of PTM loci, such as algorithm optimization. Secondly, other biological information may help improve the prediction of SUMOylation sites, such as protein secondary structure [16], which could be adopted in our future work. Thirdly, a model with feature fusion may obtain better performance in PTM site prediction than a model with a single feature [74]. For embedded tensors generated by different features, deep learning provides different high-level semantic representations. Hence, it is expected that the potential deficiency of a single feature can be compensated for by combining different feature representations, resulting in the realization of better feature representation for predicting SUMOylation sites. In summary, we constructed a novel residual learning-based architecture for predicting human SUMOylation sites with great effectiveness and robustness, and it has the potential to be applied broadly to predicting other types of modification sites.

## Figures and Tables

**Table 1:** Comparison of performance of the different models for prediction of SUMOylation sites based on five-fold cross-validation *.

Model	*Sn*	*Sp*	*MCC*	*ACC*	AUC
RF_AAindex [15]	0.697 ± 0.004	0.650 ± 0.000	0.348 ± 0.004	0.674 ± 0.002	0.742 ± 0.003
RF_BLOSUM62 [15]	0.664 ± 0.004	0.650 ± 0.000	0.314 ± 0.004	0.657 ± 0.002	0.722 ± 0.003
RF_EAAC [15]	0.693 ± 0.008	0.650 ± 0.000	0.344 ± 0.008	0.672 ± 0.004	0.738 ± 0.005
RF_ZScale [15]	0.661 ± 0.006	0.650 ± 0.000	0.311 ± 0.006	0.655 ± 0.003	0.721 ± 0.004
LGBM_AAindex [39]	0.709 ± 0.011	0.650 ± 0.000	0.360 ± 0.011	0.680 ± 0.005	0.752 ± 0.003
LGBM_BLOSUM62 [39]	0.708 ± 0.013	0.650 ± 0.000	0.358 ± 0.013	0.679 ± 0.007	0.748 ± 0.005
LGBM_EAAC [39]	0.734 ± 0.008	0.650 ± 0.000	0.385 ± 0.008	0.692 ± 0.004	0.762 ± 0.004
LGBM_ZScale [39]	0.694 ± 0.012	0.650 ± 0.000	0.344 ± 0.012	0.672 ± 0.006	0.741 ± 0.005
CNN_AAindex [31]	0.784 ± 0.007	0.650 ± 0.000	0.438 ± 0.008	0.717 ± 0.004	0.788 ± 0.005
CNN_BLOSUM62 [31]	0.784 ± 0.006	0.650 ± 0.000	0.438 ± 0.007	0.717 ± 0.003	0.788 ± 0.004
CNN_EAAC [31]	0.771 ± 0.009	0.650 ± 0.000	0.424 ± 0.010	0.711 ± 0.004	0.782 ± 0.004
CNN_ZScale [31]	0.779 ± 0.008	0.650 ± 0.000	0.433 ± 0.009	0.714 ± 0.004	0.785 ± 0.005
RSCNN_AAindex [63]	0.803 ± 0.008	0.650 ± 0.000	0.458 ± 0.009	0.726 ± 0.004	0.799 ± 0.004
RSCNN_BLOSUM62 [63]	0.803 ± 0.007	0.650 ± 0.000	0.458 ± 0.008	0.726 ± 0.004	0.799 ± 0.003
RSCNN_EAAC [63]	0.763 ± 0.007	0.650 ± 0.000	0.416 ± 0.008	0.706 ± 0.004	0.774 ± 0.004
RSCNN_ZScale [63]	0.802 ± 0.007	0.650 ± 0.000	0.457 ± 0.007	0.726 ± 0.003	0.800 ± 0.004

* The model name is a combination of the algorithm and feature names. For example, RF_AAindex combines the RF algorithm and the AAindex feature. The abbreviations of the algorithms and features are described in “Materials and Methods”. Each measure (e.g., *Sn*, *Sp*, *MCC*, *ACC*, and AUC) is shown as the average ± standard deviation of the corresponding values of the models trained and evaluated based on five-fold cross-validation. The *Sp* values were fixed to allow fair comparison of the *Sn*, *MCC*, and *ACC* measures across different models.

**Table 2:** Comparison of performance between ResSUMO and the reported models based on five-fold cross-validation and independent tests.

Model	*Sn*	*Sp*	*MCC*	*ACC*	AUC
	Five-fold cross-validation				
SUMO-Forest	0.729 ± 0.006	0.650 ± 0.000	0.380 ± 0.006	0.689 ± 0.003	0.760 ± 0.003
iSUMOK-PseAAC	0.506 ± 0.023	0.650 ± 0.000	0.158 ± 0.023	0.578 ± 0.012	0.620 ± 0.016
ResSUMO	0.802 ± 0.007	0.650 ±0.000	0.457 ± 0.007	0.726 ± 0.003	0.800 ± 0.004
	Independent test				
SUMO-Forest	0.745 ± 0.002	0.650 ± 0.000	0.397 ± 0.002	0.698 ± 0.001	0.769 ± 0.002
iSUMOK-PseAAC	0.524 ± 0.006	0.650 ± 0.000	0.176 ± 0.006	0.587 ± 0.003	0.628 ± 0.006
ResSUMO	0.795 ± 0.007	0.650 ±0.000	0.450 ± 0.008	0.722 ± 0.003	0.801 ± 0.003

**Table 3:** Performance of ResSUMO and the CNN models with different numbers of convolution layers in five-fold cross-validation.

Model *	*Sn*	*Sp*	*MCC*	*ACC*	AUC
CNN-1	0.716 ± 0.015	0.650 ± 0.000	0.367 ± 0.015	0.683 ± 0.007	0.747 ± 0.009
CNN-2	0.779 ± 0.008	0.650 ± 0.000	0.433 ± 0.009	0.714 ± 0.004	0.785 ± 0.005
CNN-4	0.773 ± 0.005	0.650 ± 0.000	0.426 ± 0.006	0.711 ± 0.003	0.783 ± 0.002
CNN-6	0.765 ± 0.009	0.650 ± 0.000	0.418 ± 0.009	0.708 ± 0.004	0.779 ± 0.007
CNN-8	0.764 ± 0.013	0.650 ± 0.000	0.417 ± 0.014	0.707 ± 0.006	0.778 ± 0.005
ResSUMO	0.802 ± 0.007	0.650 ± 0.000	0.457 ± 0.007	0.726 ± 0.003	0.800 ± 0.004

* The number in each CNN model name represents the number of convolutional layers.

## Data Availability

All data used in this study were already publicly available via http://bioinfogo.org/ResSUMO/ (accessed on 17 July 2022). The code and the data are available via https://github.com/zhuyaf521/ResSUMO (accessed on 17 July 2022).

## Supplementary Materials

The following supporting information can be downloaded at https://www.mdpi.com/article/10.3390/cells11172646/s1. Figure S1: The performance of the RF_EAAC classifier was constructed using different window sizes through the five-fold cross-validation. Window size of 39 highlighted by the red spot was selected as the peptide length for the classifier construction in this study; Figure S2: An illustrated example of the EAAC descriptor; Figure S3: The BLOSUM62 amino acid substitution matrix [33]; Figure S4: An illustrated example of the architecture of deep learning for the CNN model used the BLOSUM62 encoding approach as the characteristic matrix of the input layer; Figure S5: The training and validation accuracy and loss curves of the ResSUMO model for five-fold cross-validation. The training curves were colored orange, and the validation curves were colored blue; Figure S6: The AUC values of ResSUMO of different classifiers in terms of five-fold cross-validation; Figure S7: Performance comparison of the 16 models in the independent test; Figure S8: Performance comparison of each machine-learning model (A) or deep-learning model (B) in five-fold cross-validation and independent test; Figure S9: Sequence pattern surrounding the SUMOylation sites, including the significantly enriched and depleted residues, using the original dataset for the construction of SUMO-Forest [18] that included 755 positives (above) and 9944 negatives (below) (A), and the dataset for iSUMOK-PseAAC [20] that included 4987 positives and 5000 negatives (B). P < 0.05, student’s *t*-test with Bonferroni correction. Table S1: A comprehensive summary of the reported classifiers for predicting SUMOylation sites; Table S2: The experimental data used in this study were derived from three literature and one database; Table S3: Summary of the 14 types of physicochemical properties of amino acids. For each property, there is a set of 20 numerical values for all amino acids; Table S4: ZScale for the 20 amino acids; Table S5: The AUC and PRC values are generated by the iLearnPlus-Estimator module in terms of 5-fold cross-validation; Table S6: Performances of different models for predicting SUMOylation sites on the independent test; Table S7: Performance comparison of the original models and reproduced models; Table S8: The performances of the reproduced models on our independent test dataset#.
