# Supplementary material for: ResSUMO: A Deep Learning Architecture Based on Residual Structure for Prediction of Lysine SUMOylation Sites
Source: Cells. 2022 Aug 25;11(17):2646. doi: 10.3390/cells11172646 (PMC9454673; doi:10.3390/cells11172646)
Supplement: Supplementary file 1 [file cells-11-02646-s001.zip › cells-1858212-supplementary.pdf]

ResSUMO: A Deep Learning Architecture Based on Residual Structure for Prediction of Lysine SUMOylation Sites

Yafei Zhu[1], Yuhai Liu[2], Yu Chen[1], Lei Li[3,1*]

Table S1. A comprehensive summary of the reported classifiers for predicting SUMOylation sites.

Tool	Algorithm*	Encoding scheme	Evaluation strategy	Benchmark Dataset (positives/negatives)	Balanced Samples?	Web Server	Code availability	Window size	Published year	PMID
pSumo-CD	CD	PseAAC	cross-validation	755/9,944	No	No	No	21	2016	27354696
iAcet-Sumo	SVM	One-Hot	cross-validation	5,963/13,743	No	No	No	21	2018	30015011
SUMOgo	SVM	BE; AAindex; Structural features	independent test	1,166/2,332	Yes	No	No	21	2018	30341374
SumSec	Bagging C4.5 DT	SSpre-occur; SSpre-bigram	cross-validation	780/780	Yes	No	No	31	2018	30544729
HseSUMO	DT	HSE	cross-validation	780/780	Yes	No	No	31	2019	30999862
SUMO-Forest	Cascade Forest	SP; BK	cross-validation	755/9,944	No	No	Yes	21	2020	32160959
C-iSUMO	AdaBoost DT	ASA; Torsion Angles	cross-validation	780/780	Yes	No	No	31	2020	32604027
iSUMOK-PseAAC	ANN	PseAAC; SVV; SM;FV; PRIM; RPRIM; AAPIV; RAAPIV	Independent test	4,987/5,000	Yes	No	Yes	41	2021	34430072

* CD: covariant discriminant; SVM: support vector machine; DT: decision tree; ANN: artificial neural network; PseAAC: pseudo-position specific scoring matrix; AAindex: a database of amino acid indices and amino acid mutation matrices; SSpre-occur and SSpre-bigram: predicted secondary structure occurrence and profile-bigram; HSE: half-sphere exposure; SP: statistics property; BK: bi-gram and k-skip-bi-gram; ASA: accessible surface area; SVV: site vicinity vector; SM: statistical moments; FV: frequency vector; PRIM: position relative incidence matrix; RPRIM: reverse position relative incidence matrix; AAPIV: accumulative absolute position incidence vector; RAAPIV: reverse accumulative absolute position incidence vector; RSCNN: The residual structure layered CNN architecture.

Table S2. The experimental data used in this study were derived from three literature and one database.

Source	Species	Number of SUMOylation sites	Published year	PMID
PLMD database	Human	7820	2017	28529077
literature	Human	40765	2017	28112733
literature	Human	14869	2018	29942033
literature	Human	4987	2021	34430072

Table S3. Summary of the 14 types of physicochemical properties of amino acids. For each property, there is a set of 20 numerical values for all amino acids.

Accession number	A	R	N	D	C	Q	E	G	H	I	L	K	M	F	P	S	T	W	Y	V
FAUJ830101	0.31	-1.01	-0.60	-0.77	1.54	-0.22	-0.64	0.00	0.13	1.80	1.70	-0.99	1.23	1.79	0.72	-0.04	0.26	2.25	0.96	1.22
FINA910104	1.	1.70	1.	0.70	1.	1.	0.70	1.50	1.	1.	1.	1.70	1.	1.	0.10	1.	1.	1.	1.	1.
GARJ730101	0.28	0.10	0.25	0.21	0.28	0.35	0.33	0.17	0.21	0.82	1.00	0.09	0.74	2.18	0.39	0.12	0.21	5.70	1.26	0.60
GUYH850101	0.10	1.91	0.48	0.78	-1.42	0.95	0.83	0.33	-0.50	-1.13	-1.18	1.40	-1.59	-2.12	0.73	0.52	0.07	-0.51	-0.21	-1.27
LEVM760101	-0.5	3.0	0.2	2.5	-1.0	0.2	2.5	0.0	-0.5	-1.8	-1.8	3.0	-1.3	-2.5	-1.4	0.3	-0.4	-3.4	-2.3	-1.5
RADA880108	-0.06	-0.84	-0.48	-0.80	1.36	-0.73	-0.77	-0.41	0.49	1.31	1.21	-1.18	1.27	1.27	0.	-0.50	-0.27	0.88	0.33	1.09
FINA910102	1.	0.70	1.	1.70	1.	1.	1.70	1.30	1.	1.	1.	0.70	1.	1.	13.	1.	1.	1.	1.	1.
OLSK800101	1.38	0.00	0.37	0.52	1.43	0.22	0.71	1.34	0.66	2.32	1.47	0.15	1.78	1.72	0.85	0.86	0.89	0.82	0.47	1.99
KIDA850101	-0.27	1.87	0.81	0.81	-1.05	1.10	1.17	-0.16	0.28	-0.77	-1.10	1.70	-0.73	-1.43	-0.75	0.42	0.63	-1.57	-0.56	-0.40
NADH010102	51	-144	-84	-78	137	-128	-115	-13	-55	106	103	-205	73	108	-79	-26	-3	69	11	108
JANJ780101	27.8	94.7	60.1	60.6	15.5	68.7	68.2	24.5	50.7	22.8	27.6	103.0	33.5	25.5	51.5	42.0	45.0	34.7	55.2	23.7
ZIMJ680104	6.00	10.76	5.41	2.77	5.05	5.65	3.22	5.97	7.59	6.02	5.98	9.74	5.74	5.48	6.30	5.68	5.66	5.89	5.66	5.96
JANJ780103	15.	67.	49.	50.	5.	56.	55.	10.	34.	13.	16.	85.	20.	10.	45.	32.	32.	17.	41.	14.
NADH010103	41	-109	-74	-47	169	-104	-90	-18	-35	104	103	-148	77	128	-81	-31	10	102	36	116

Table S4. ZScale for the 20 amino acids.

Amino acid	Z1	Z2	Z3	Z4	Z5
A	0.24	-2.32	0.60	-0.14	1.30
C	0.84	-1.67	3.71	0.18	-2.65
D	3.98	0.93	1.93	-2.46	0.75
E	3.11	0.26	-0.11	-3.04	-0.25
F	-4.22	1.94	1.06	0.54	-0.62
G	2.05	4.06	0.36	-0.82	-0.38
H	2.47	1.95	0.26	3.90	0.09
I	-3.89	-1.73	-1.71	-0.84	0.26
K	2.29	0.89	-2.49	1.49	0.31
L	-4.28	-1.30	-1.49	-0.72	0.84
M	-2.85	-0.22	0.47	1.94	-0.98
N	3.05	1.60	1.04	-1.15	1.61
P	-1.66	0.27	1.84	0.70	2.00
Q	1.75	0.50	-1.44	-1.34	0.66
R	3.52	2.50	-3.50	1.99	-0.17
S	2.39	-1.07	1.15	-1.39	0.67
T	0.75	-2.18	-1.12	-1.46	-0.40
V	-2.59	-2.64	-1.54	-0.85	-0.02
W	-4.36	3.94	0.59	3.44	-1.59
Y	-2.54	2.44	0.43	0.04	-1.47

The property of each amino acid can be represented by five ZScale values [1].

Table S5. The AUC and PRC values are generated by the iLearnPlus-Estimator module in terms of 5-fold cross-validation.

Feature*	AUC	PRC
EAAC	0.749	0.727
AAindex	0.737	0.721
BLOSUM62	0.736	0.721
ZScale	0.731	0.717
OH	0.718	0.704
EGAAC	0.717	0.697
CKSAAP	0.691	0.673
DDE	0.654	0.638
DPC	0.653	0.637
AAC	0.651	0.624
PAAC	0.650	0.622

* EAAC: Enhanced Amino Acid Composition; AAindex: Amino Acid indices; BLOSUM62: BLOcks SUbstitution Matrix 62; OH: One Hot; EGAAC: Enhanced Grouped Amino Acid Composition; CKSAAP: Enhanced Grouped Amino Acid Composition; DDE: Dipeptide Deviation from Expected Mean; DPC: Di-Peptide Composition; AAC: Amino Acid Composition; PAAC: Pseudo-Amino Acid Composition

Table S6. Performances of different models for predicting SUMOylation sites on the independent test.

Model	Sn	Sp	MCC	ACC	AUC
RF_AAindex	0.698 ± 0.003	0.650 ± 0.000	0.349 ± 0.003	0.674 ± 0.001	0.745 ± 0.002
RF_BLOSUM62	0.671 ± 0.005	0.650 ± 0.000	0.321 ± 0.005	0.660 ± 0.003	0.728 ± 0.001
RF_EAAC	0.694 ± 0.003	0.650 ± 0.000	0.345 ± 0.003	0.672 ± 0.002	0.741 ± 0.002
RF_ZScale	0.669 ± 0.005	0.650 ± 0.000	0.319 ± 0.005	0.660 ± 0.002	0.726 ± 0.001
LGBM_AAindex	0.719 ± 0.005	0.650 ± 0.000	0.369 ± 0.005	0.684 ± 0.003	0.756 ± 0.002
LGBM_BLOSUM62	0.712 ± 0.003	0.650 ± 0.000	0.362 ± 0.003	0.681 ± 0.002	0.755 ± 0.001
LGBM_EAAC	0.741 ± 0.006	0.650 ± 0.000	0.393 ± 0.006	0.696 ± 0.003	0.765 ± 0.002
LGBM_ZScale	0.701 ± 0.006	0.650 ± 0.000	0.351 ± 0.006	0.675 ± 0.003	0.745 ± 0.003
CNN_AAindex	0.777 ± 0.009	0.650 ± 0.000	0.431 ± 0.009	0.714 ± 0.004	0.790 ± 0.003
CNN_BLOSUM62	0.782 ± 0.007	0.650 ± 0.000	0.436 ± 0.007	0.716 ± 0.003	0.789 ± 0.001
CNN_EAAC	0.779 ± 0.004	0.650 ± 0.000	0.432 ± 0.004	0.714 ± 0.002	0.785 ± 0.001
CNN_ZScale	0.771 ± 0.004	0.650 ± 0.000	0.424 ± 0.005	0.711 ± 0.002	0.786 ± 0.002
RSCNN_AAindex	0.792 ± 0.005	0.650 ±0.000	0.447 ± 0.005	0.721 ± 0.002	0.801 ± 0.003
RSCNN_BLOSUM62	0.794 ± 0.006	0.650 ±0.000	0.448 ± 0.006	0.722 ± 0.003	0.801 ± 0.003
RSCNN_EAAC	0.755 ± 0.007	0.650 ±0.000	0.408 ± 0.008	0.703 ± 0.004	0.773 ± 0.002
RSCNN_ZScale	0.795 ± 0.007	0.650 ±0.000	0.450 ± 0.008	0.722 ± 0.003	0.801 ± 0.003

Table S7. Performance comparison of the original models and reproduced models.

Model	Sn	Sp	MCC	ACC	AUC
SUMO-Forest (original)	92.05	99.03	98.54	89.15	99.05
SUMO-Forest*	95.36	98.13	97.93	86.00	98.96
ISUMOK-PseAAC (original)	94.51	94.24	94.79	89.03	96.09
ISUMOK-PseAAC*	93.72	93.46	93.59	87.19	96.10

The performances of the original SUMO-Forest and ISUMOK-PseAAC were extracted from literature [2, 3]. The models marked by * were reproduced according to the literature.

Table S8. The performances of the reproduced models on our independent test dataset#.

Model	Sn	Sp	MCC	ACC	AUC
SUMO-Forest*	8.95	95.07	52.01	7.94	52.55
ISUMOK-PseAAC*	56.27	55.30	55.78	11.57	57.47

#The independent test dataset contained 3,728 SUMOylation sites and 3,728 non-SUMOylation sites.

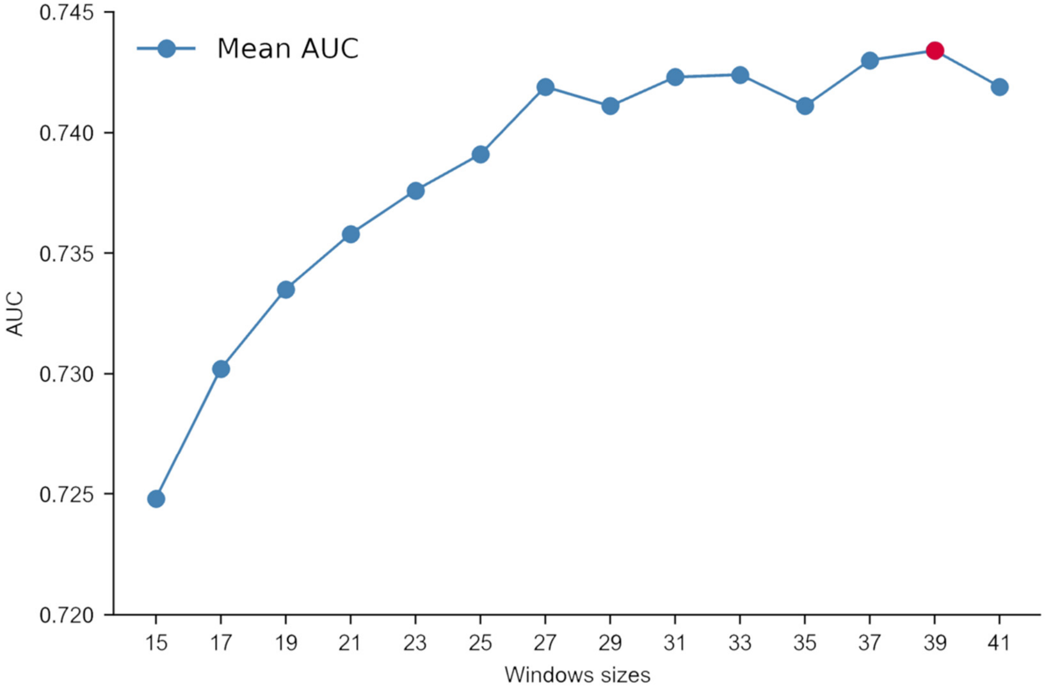

Figure S1. The performance of the RF_EAAC classifier was constructed using different window sizes through the five-fold cross-validation. Window size of 39 highlighted by the red spot was selected as the peptide length for the classifier construction in this study.

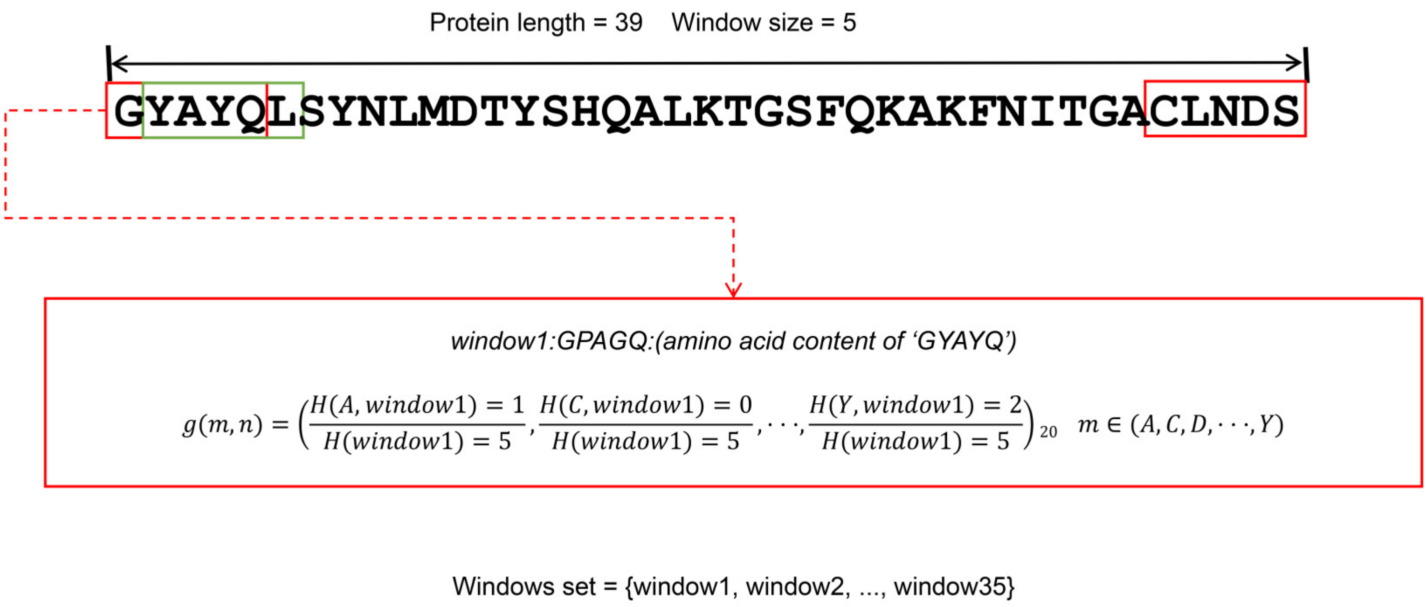

Figure S2. An illustrated example of the EAAC descriptor.

	A	R	N	D	C	Q	E	G	H	I	L	K	M	F	P	S	T	W	Y	V
**A**	4																			
R	-1	5																		
N	-2	0	6																	
D	-2	-2	1	6																
C	0	-3	-3	-3	9															
Q	-1	1	0	0	-3	5														
E	-1	0	0	2	-4	2	5													
G	0	-2	0	-1	-3	-2	-2	6												
H	-2	0	1	-1	-3	0	0	-2	8											
I	-1	-3	-3	-3	-1	-3	-3	-4	-3	4										
L	-1	-2	-3	-4	-1	-2	-3	-4	-3	2	4									
K	-1	2	0	-1	-3	1	1	-2	-1	-3	-2	5								
M	-1	-1	-2	-3	-1	0	-2	-3	-2	1	2	-1	5							
F	-2	-3	-3	-3	-2	-3	-3	-3	-1	0	0	-3	0	6						
P	-1	-2	-2	-1	-3	-1	-1	-2	-2	-3	-3	-1	-2	-4	7					
S	1	-1	1	0	-1	0	0	0	-1	-2	-2	0	-1	-2	-1	4				
T	0	-1	0	-1	-1	-1	-1	-2	-2	-1	-1	-1	-1	-2	-1	1	5			
W	-3	-3	-4	-4	-2	-2	-3	-2	-2	-3	-2	-3	-1	1	-4	-3	-2	11		
Y	-2	-2	-2	-3	-2	-1	-2	-3	2	-1	-1	-2	-1	3	-3	-2	-2	2	7	
V	0	-3	-3	-3	-1	-2	-2	-3	-3	3	1	-2	1	-1	-2	-2	0	-3	-1	4

Figure S3. The BLOSUM62 amino acid substitution matrix [4].

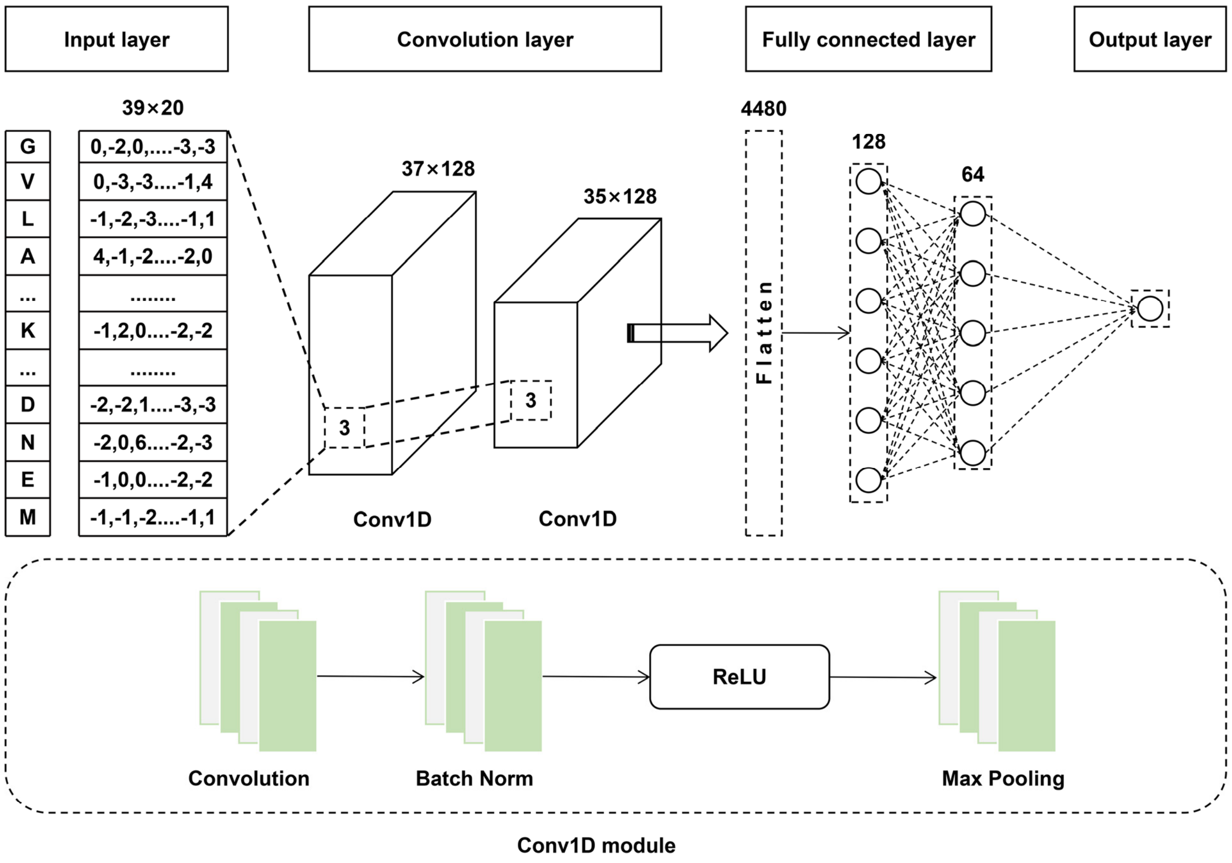

Figure S4. An illustrated example of the architecture of deep learning for the CNN model used the BLOSUM62 encoding approach as the characteristic matrix of the input layer.

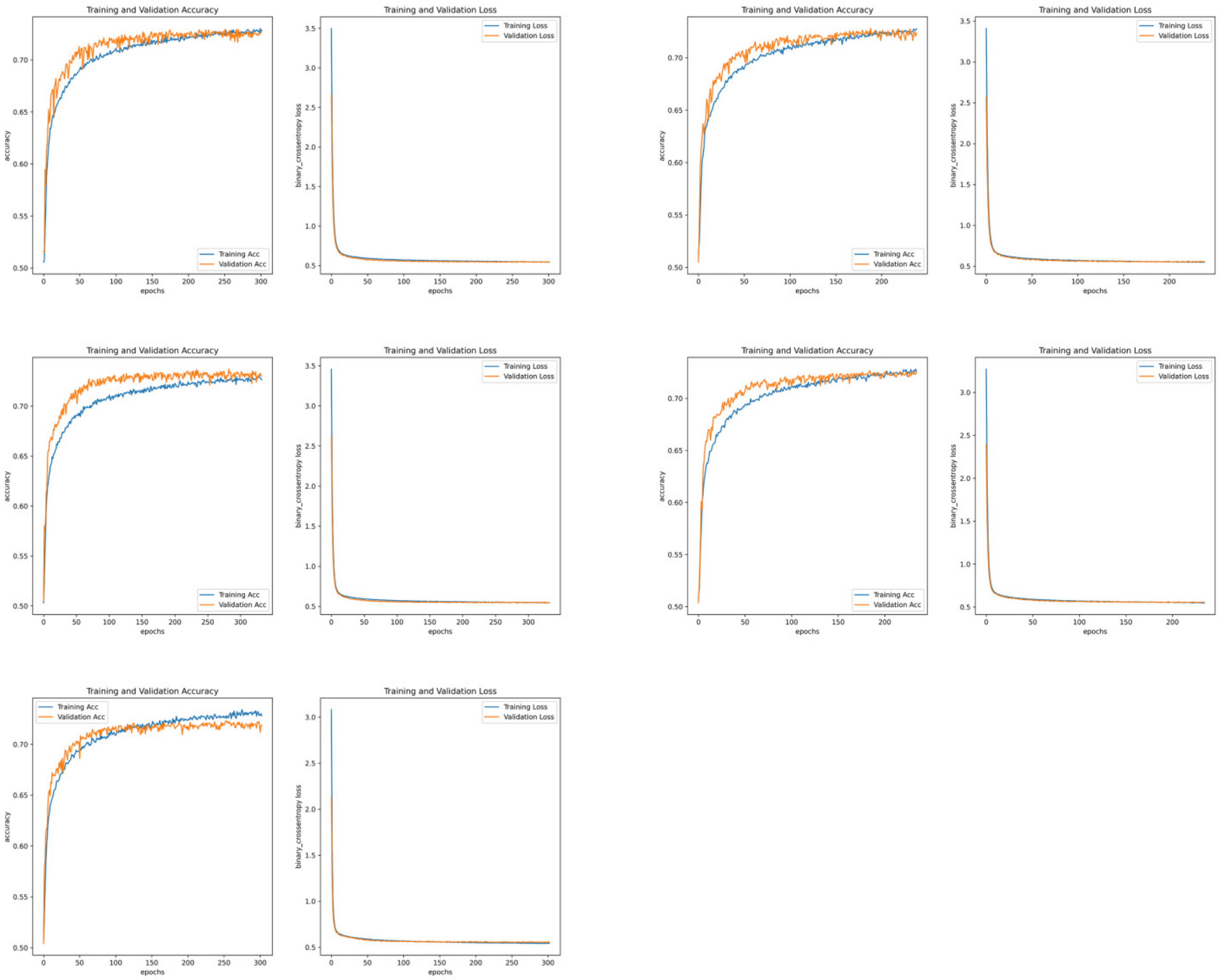

Figure S5. The training and validation accuracy and loss curves of the ResSUMO model for five-fold cross-validation. The training curves were colored orange, and the validation curves were colored blue.

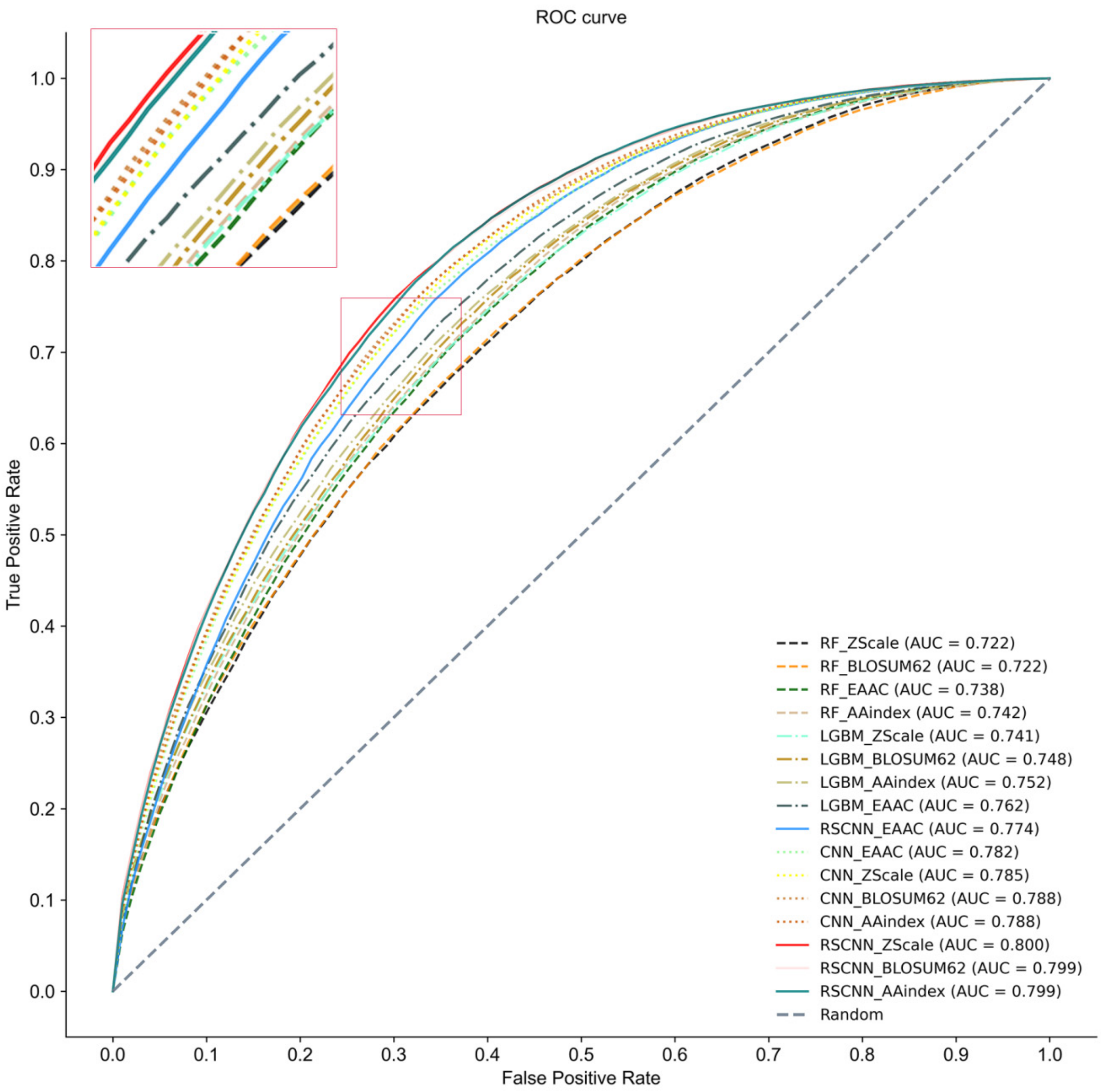

Figure S6. The AUC values of ResSUMO of different classifiers in terms of five-fold cross-validation.

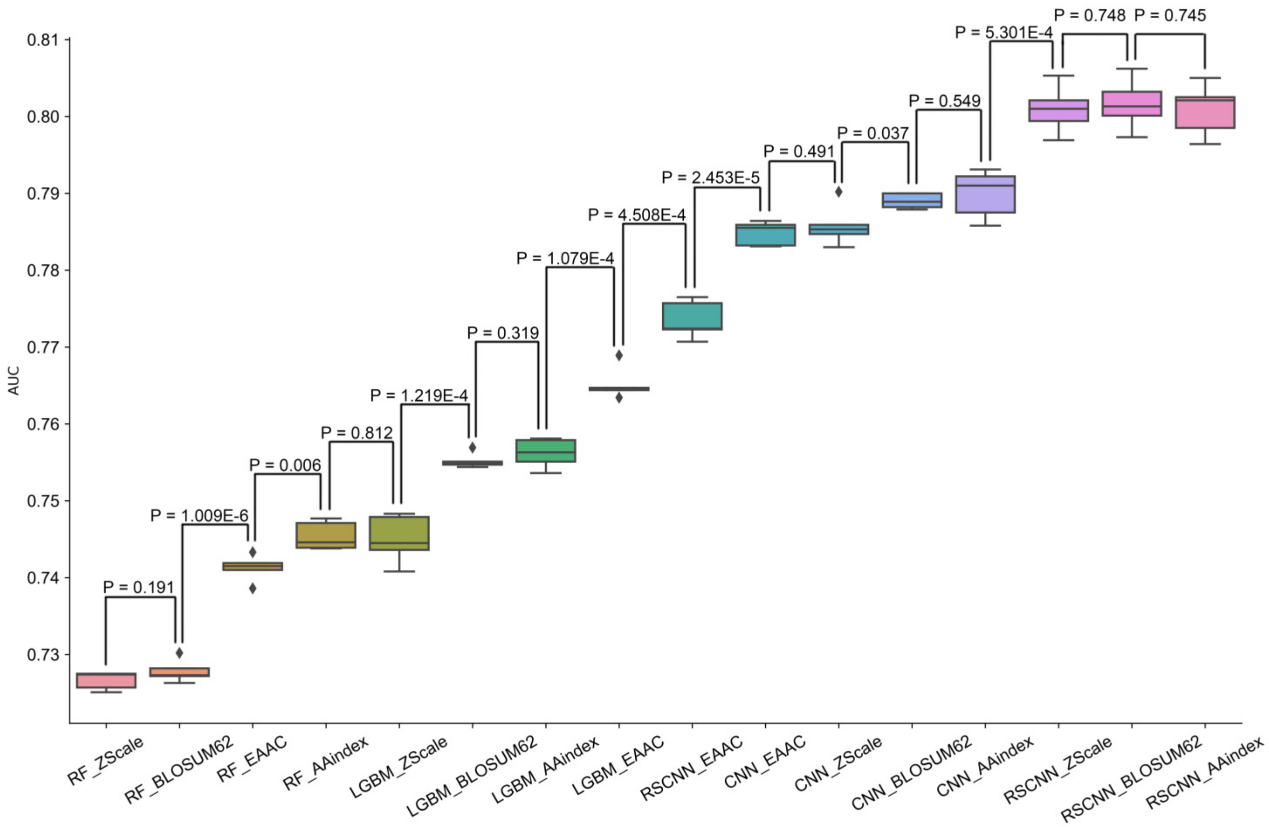

Figure S7. Performance comparison of the 16 models in the independent test.

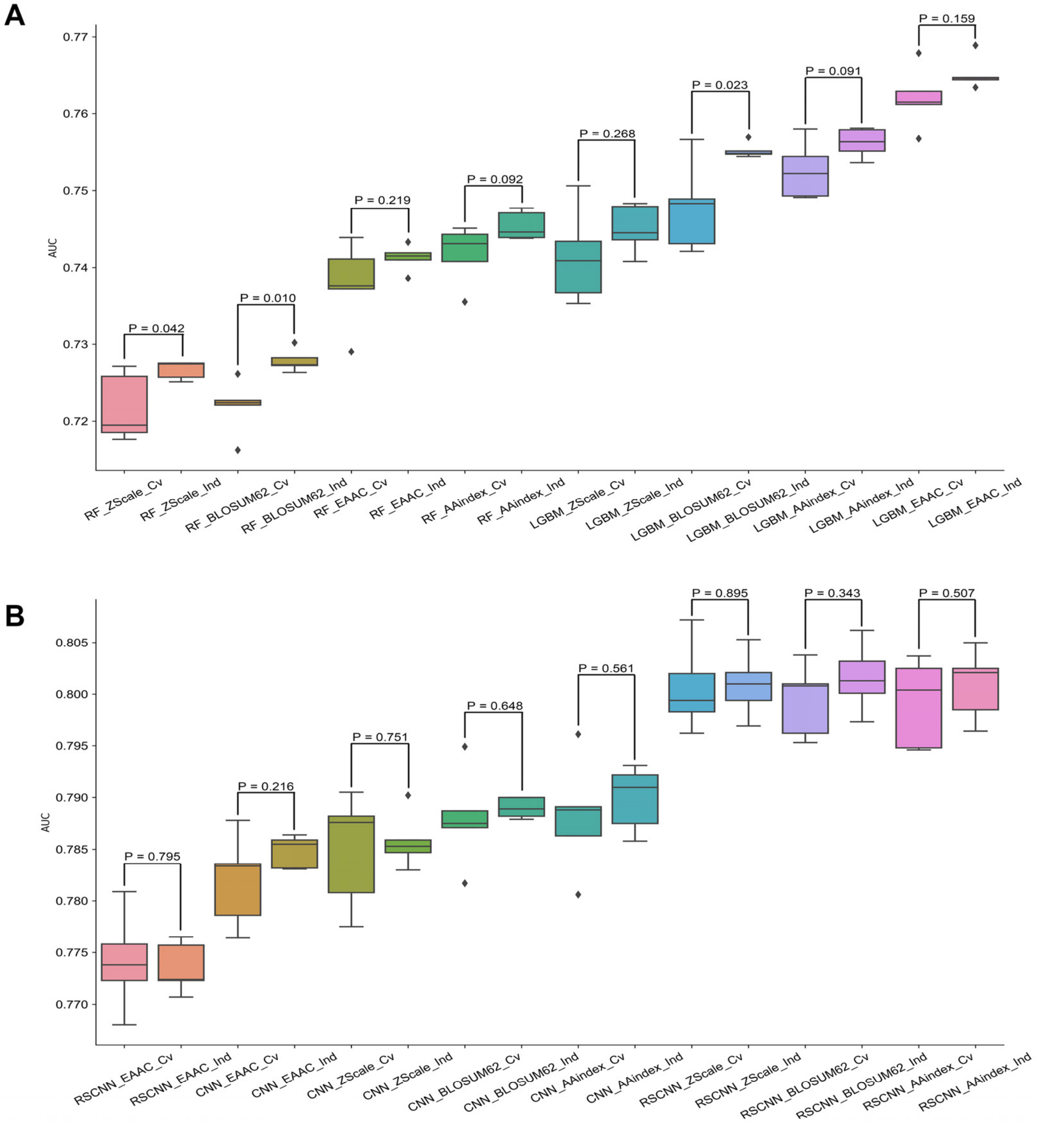

Figure S8. Performance comparison of each machine-learning model (A) or deep-learning model (B) in five-fold cross-validation and independent test.

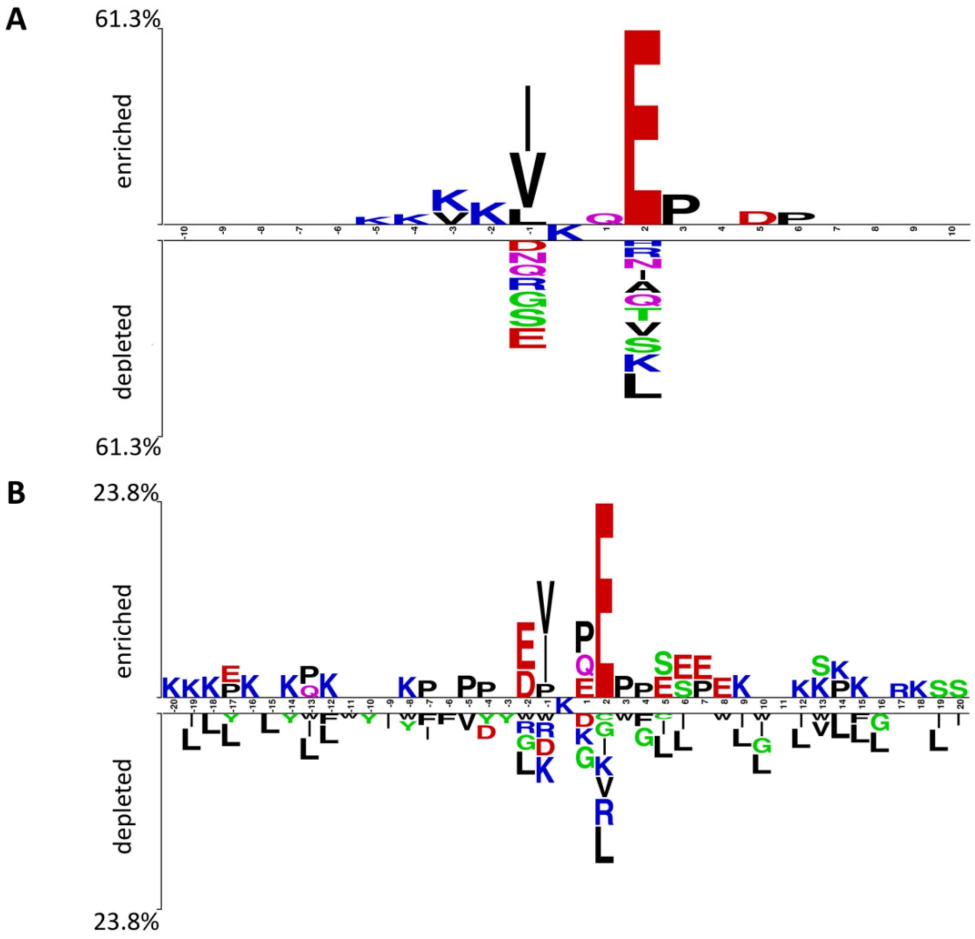

Figure S9. Sequence pattern surrounding the SUMOylation sites, including the significantly enriched and depleted residues, using the original dataset for the construction of SUMO-Forest [3] that included 755 positives (above) and 9,944 negatives (below) (A), and the dataset for iSUMOK-PseAAC [2] that included 4,987 positives and 5,000 negatives (B). $P < 0.05$, student's T-test with Bonferroni correction.

References:

1. Chen Z, Zhao P, Li F et al. iFeature: a Python package and web server for features extraction and selection from protein and peptide sequences, Bioinformatics 2018;34:2499-2502.
2. Khan YD, Khan NS, Naseer S et al. iSUMOK-PseAAC: prediction of lysine sumoylation sites using statistical moments and Chou's PseAAC, PeerJ 2021;9:e11581.
3. Qian Y, Ye S, Zhang Y et al. SUMO-Forest: A Cascade Forest based method for the prediction of SUMOylation sites on imbalanced data, Gene 2020;741:144536.
4. Pearson WR. Selecting the Right Similarity-Scoring Matrix, Curr Protoc Bioinformatics 2013;43:3 5 1-3 5 9.
5. Chen Z, Zhao P, Li C et al. iLearnPlus: a comprehensive and automated machine-learning platform for nucleic acid and protein sequence analysis, prediction and visualization, Nucleic Acids Res 2021;49:e60.
